# Molecular Physiology of the Neuronal Synapse

**DOI:** 10.3390/cimb48010088

**Published:** 2026-01-15

**Authors:** María Jesús Ramírez-Expósito, Cristina Cueto-Ureña, José Manuel Martínez-Martos

**Affiliations:** Experimental and Clinical Physiopathology Research Group CTS-1039, Department of Health Sciences, School of Health Sciences, University of Jaén, 23071 Jaén, Spain; mramirez@ujaen.es (M.J.R.-E.); ccueto@ujaen.es (C.C.-U.)

**Keywords:** synaptic plasticity, synaptic homeostasis, active zone, postsynaptic density, SNARE complex, neurotransmitter release, vesicle recycling, ionotropic receptors, synaptogenesis, microglial pruning, molecular diversity

## Abstract

Neuronal synapses are the functional units of communication in the central nervous system. This review describes the molecular mechanisms regulating synaptic transmission, plasticity, and circuit refinement. At the presynaptic active zone, scaffolding proteins including bassoon, piccolo, RIMs, and munc13 organize vesicle priming and the localization of voltage-gated calcium channels. Neurotransmitter release is mediated by the SNARE complex, comprising syntaxin-1, SNAP25, and synaptobrevin, and triggered by the calcium sensor synaptotagmin-1. Following exocytosis, synaptic vesicles are recovered through clathrin-mediated, ultrafast, bulk, or kiss-and-run endocytic pathways. Postsynaptically, the postsynaptic density (PSD) serves as a protein hub where scaffolds such as PSD-95, shank, homer, and gephyrin anchor excitatory (AMPA, NMDA) and inhibitory (GABA-A, Glycine) receptors are observed. Synaptic strength is modified during long-term potentiation (LTP) and depression (LTD) through signaling cascades involving kinases like CaMKII, PKA, and PKC, or phosphatases such as PP1 and calcineurin. These pathways regulate receptor trafficking, Arc-mediated endocytosis, and actin-dependent remodeling of dendritic spines. Additionally, synapse formation and elimination are guided by cell adhesion molecules, including neurexins and neuroligins, and by microglial pruning via the complement cascade (C1q, C3) and “don’t eat me” signals like CD47. Molecular diversity is further expanded by alternative splicing and post-translational modifications. A unified model of synaptic homeostasis is required to understand the basis of neuropsychiatric and neurological disorders.

## 1. Introduction

Neuronal synapses represent the units of communication within the central nervous system, mediating rapid and precise information transfer between neurons [1,2]. These specialized intercellular junctions are not merely passive conduits but actively process and transform information during transmission, contributing significantly to the physiology of neural circuits. The complex architecture of a synapse, characterized by its asymmetric organization, enables unidirectional signal propagation from the presynaptic terminal to the postsynaptic neuron [2,3,4]. The diversity observed in the properties of synapses cannot be fully predicted solely from their ultrastructure or anatomical location, highlighting the influence of their molecular composition [1,4].

The molecular landscape of a synapse is complex, comprising thousands of distinct proteins that collectively dictate its functional specializations [4]. Recently, it has been shown that synaptic proteome diversity is specifically shaped by the levels of glutamate receptors and their associated regulatory proteins, allowing for a molecular classification that extends beyond connectivity [5]. This molecular diversity allows for many synapse types, each tailored to specific roles within neural circuits. The unique properties of individual synapses are regulated through a continuous interplay of bidirectional signals exchanged between the presynaptic and postsynaptic compartments [3,4]. Beyond the rapid, point-to-point communication facilitated by chemical synapses, neurons also engage in slower, non-synaptic signaling via neuromodulators such as neuropeptides, monoamines, acetylcholine, endocannabinoids, retinoic acid, and nitric oxide [1]. In aminergic systems, this complexity is further expanded by ‘matrix pharmacology,’ a concept describing the modulation of multiple transporters. For instance, compounds such as noribogaine inhibit the organic cation transporter 2 (OCT2) and act as synaptic reuptake inhibitors (SynRIs). These compounds exert dual inhibition on the serotonin transporter (SERT) and the vesicular monoamine transporter 2 (VMAT2), with noribogaine also inducing partial serotonin release from synaptic vesicles, thereby regulating neurotransmitter availability in both the cytosol and the vesicle [6]. These neuromodulatory signals add to synaptic information processing, contributing to the dynamic and adaptive nature of neural circuits. The inherent molecular complexity and dynamic regulation of synapses are relevant to cognitive functions observed in vertebrates, including associative learning and visual discrimination [7,8]. This molecular machinery supports the brain’s capacity for complex behaviors and adaptability.

The foundational understanding of synaptic function originated from classical physiological investigations in the mid-20th century. Pioneering work by researchers such as Bernard Katz and John Eccles characterized the fundamental mechanisms of synaptic transmission through elegant electrophysiological studies [2]. These early discoveries illuminated the central importance of synapses in brain function, laying the groundwork for subsequent molecular explorations. The transition from a purely electrophysiological perspective to a molecular understanding marked an advance in neuroscience.

A pivotal shift occurred with the elucidation of the molecular composition of synapses, which provided the necessary framework to comprehend synaptic transmission beyond its electrical properties. In the early 2000s, major advances occurred with the discovery that the synapse proteome is complex, comprising over a thousand conserved proteins, particularly within excitatory synapses [9]. This revelation altered prevailing concepts of synaptic molecular function and highlighted the critical involvement of synapses in various neurological and psychiatric disorders [10]. The identification of large multiprotein complexes, such as those associated with N-methyl-D-aspartate (NMDA) receptors and membrane-associated guanylate kinase (MAGUK) proteins, expanded the known synaptic proteome by tenfold, opening new avenues for research into synaptic organization and disease [10]. This progression from macroscopic physiological observations to detailed molecular inventories has been essential for understanding the mechanisms that govern synaptic function and plasticity.

Here, we synthesize current knowledge regarding the molecular mechanisms underlying key synaptic processes. This includes the examination of presynaptic neurotransmitter release and vesicle recycling, the organization and dynamic regulation of postsynaptic receptors, and the molecular underpinnings of synaptic plasticity, including long-term potentiation (LTP) and long-term depression (LTD). We also explore the molecular cues and cellular mechanisms governing synapse formation, maturation, and elimination, addressing the molecular diversity and specialization of synapses and the contributions of alternative splicing and post-translational modifications.

## 2. Presynaptic Molecular Mechanisms

### 2.1. Active Zone Organization and Dynamics

**Figure 1:**
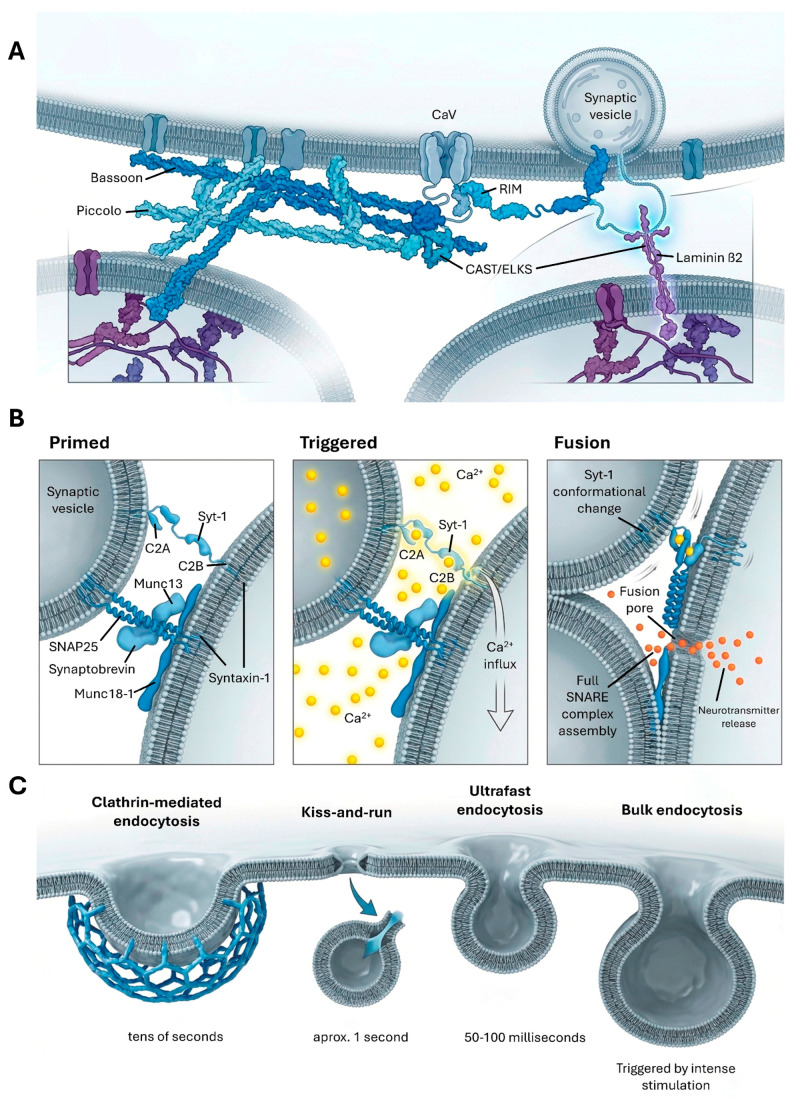
Molecular architecture of the presynaptic terminal and the vesicle cycle. (**A**) Organization of the active zone (AZ) highlighting the cytomatrix (CAZ) proteins Bassoon, Piccolo, RIM, and CAST/ELKS. The complex anchors voltage-gated calcium channels (CaV) in alignment with postsynaptic laminin β2. (**B**) The synaptic vesicle fusion cycle showing the “lever” mechanism of Synaptotagmin-1 (Syt-1). Upon Ca^2+^ influx, Syt-1 undergoes a conformational change that facilitates the full zippering of the SNARE complex (Syntaxin-1, SNAP25, and Synaptobrevin). (**C**) Multiple pathways of activity-dependent synaptic vesicle recycling, including clathrin-mediated endocytosis (CME), ultrafast endocytosis (50–100 ms), kiss-and-run (~1 s), and bulk endocytosis triggered by high-intensity stimulation.

The presynaptic active zone (AZ) is a highly specialized, electron-dense region of the presynaptic membrane crucial for rapid and efficient neurotransmitter release [11,12]. The precise organization and dynamic regulation of the AZ are fundamental for the proper functioning of the nervous system, influencing processes such as development, synaptic plasticity, and susceptibility to pathology. The structural integrity and functional efficiency of active zones are maintained through a complex interplay of molecular components (Figure 1).

The organization of the AZ involves a trans-synaptic molecular mechanism that physically links the presynaptic and postsynaptic compartments. On the presynaptic side, cytosolic active zone proteins are tightly associated with the cytosolic domains of voltage-dependent calcium channels (P/Q-, N-, and L-type) [11]. This association positions the calcium channels strategically near the sites of vesicle release. On the postsynaptic side, a key synapse organizer, laminin β2, is expressed by the postsynaptic cell and specifically accumulates directly opposite the postsynaptic specialization [11]. The extracellular domains of the presynaptic calcium channels directly interact with laminin β2, anchoring the presynaptic complex in precise alignment with the postsynaptic machinery. While this specific interaction is a key organizer at the neuromuscular junction and many central excitatory synapses, current evidence suggests that the core anchoring of voltage-gated calcium channels via RIM and RIM-BP scaffolds is a universal feature across all central synapse types, ensuring fast neurotransmission regardless of the neurotransmitter identity [1,13]. It is noteworthy that while calcium influx through these channels is absolutely essential for synaptic transmission, the initial formation of the active zone itself does not necessitate this calcium influx [11]. This suggests a two-tiered regulatory system where structural assembly precedes functional activation. The direct interaction between presynaptic calcium channels and postsynaptic laminin β2 for anchoring the presynaptic complex reveals a critical trans-synaptic mechanism. This indicates that the postsynaptic neuron actively contributes to the structural organization of the presynaptic terminal, highlighting the interdependent nature of synaptic compartments and emphasizing the bidirectional communication essential for proper synapse assembly and function.

Active zones in adult synapses are not static structures; they require continuous maintenance to preserve their integrity [11]. Disruptions to AZs are observed in conditions of aging and various diseases affecting both the central and peripheral nervous systems. The number, size, and distribution of active zones profoundly influence how information is processed within a neuronal circuit and how the circuit adapts to internal or external cues [14]. Key molecular players that regulate active zone density include extracellular matrix laminins, voltage-dependent calcium channels, amyloid precursor proteins, the small GTPase Rab3, endocytosis mechanisms involving synaptojanin, and cytoskeletal proteins such as spectrins and β-adducin [14]. The maintenance of active zone density during developmental growth, particularly in the central and peripheral nervous systems, appears to be mediated by molecular mechanisms that are independent of neuronal activity. This dynamic regulation of active zone density and composition is crucial for maintaining synaptic efficiency and adaptability. The involvement of numerous proteins in their organization and maintenance points to a complex and dynamic regulatory network that ensures adaptable and sustainable neurotransmission, which is fundamental for neural network function [15].

Large scaffolding proteins, Bassoon and Piccolo, are integral components of the cytomatrix at the active zone (CAZ), the specialized region where neurotransmitter release occurs [16]. These proteins share significant sequence similarity and fulfill both overlapping and distinct functions in organizing the CAZ. Their roles include the assembly of active zones, the precise localization of voltage-gated Ca^2+^ channels near release sites, and synaptic vesicle priming [15,16]. Piccolo, in particular, also contributes to the dynamic assembly of the actin cytoskeleton at the AZ. Additional functions for Bassoon and Piccolo in shaping presynaptic composition have been revealed, including local regulation of the ubiquitin-proteasome system, where ubiquitination can affect the function of presynaptic proteins independently of their proteasomal degradation. Furthermore, these scaffolds couple activity-induced molecular rearrangements at the presynapse with the reprogramming of neuronal gene expression [15].

Rab3-interacting molecules (RIMs) and Munc13 are also essential active zone proteins, playing key roles in synaptic vesicle docking and priming [17]. These proteins, along with CAST/ELKS and Bassoon/Piccolo, form a large molecular complex in the brain, creating a central interaction node where domains of Piccolo, Bassoon, CAST, and RIM1 converge on the N-terminal of Munc13-1 [18,19]. Additionally, it has been identified in *C. elegans* cholinergic neurons, that the VAChT transporter (UNC-17) interacts with this complex to regulate tubulin acetylation through a tripartite regulatory complex (UNC-104/UNC-17/MEC-17). Disruption of this complex releases the acetyltransferase MEC-17, increasing tubulin acetylation, which compromises the processivity of the UNC-104 (KIF1A) molecular motor and reduces synaptic vesicle transport efficiency [20]. Specifically, RIM1 and Bassoon directly bind to different regions of CAST, forming a ternary complex, while Piccolo also interacts with the Bassoon-binding region of CAST [17]. The N-terminal region of Munc13-1 acts as a central hub where domains of Piccolo, Bassoon, CAST, and RIM1 converge to integrate the protein machinery at the release site [19]. Munc13 is essential for both fast and modulatory release; however, its membrane-binding C2C domain is specifically crucial for neuropeptide secretion from large dense-core vesicles (DCVs), unlike the redundant C2B domain in small synaptic vesicle (SV) exocytosis [4,21]. Furthermore, while Syt-1 is the primary sensor for synchronous SV release, DCV exocytosis relies more heavily on the high-affinity Ca^2+^ sensor Synaptotagmin-7 (Syt-7), reflecting different evolutionary requirements for kinetics and spatiotemporal precision [21] (Table 1).

### 2.2. Neurotransmitter Synthesis, Packaging, and Release

Neurotransmission, the rapid communication between neurons, fundamentally relies on the precisely regulated release of chemical neurotransmitters from presynaptic nerve terminals [22]. These neurotransmitters are synthesized within the neuron and subsequently packaged into small, spherical organelles known as synaptic vesicles (SVs) [23]. The release process, termed exocytosis, is triggered by the influx of calcium ions into the presynaptic terminal following an action potential [24]. This exocytosis occurs in three modes: synchronous (triggered by action potentials), asynchronous (with variable delay), and spontaneous (in the absence of stimuli), which share core fusion machinery but differ in calcium sensor requirements [25].

At the heart of this rapid membrane fusion process lies the soluble N-ethylmaleimide sensitive factor attachment protein receptor (SNARE) complex [22]. This complex is formed by the interaction of three key proteins: synaptobrevin (also known as VAMP2), a v-SNARE located on the synaptic vesicle membrane, and syntaxin-1 and SNAP25, which are t-SNAREs anchored to the presynaptic plasma membrane [22]. These SNARE proteins assemble into a stable four-helix bundle, a structure that physically pulls the vesicle and plasma membranes into close proximity, thereby driving membrane fusion (Table 1).

Synaptotagmin-1 (Syt1) serves as the primary calcium sensor that directly triggers neurotransmitter release [22]. Prior to calcium influx, Syt-1 is already bound to the SNARE complex, a state that inhibits the release of neurotransmitters. Calcium triggering then requires a rearrangement of the primary interface involving the dissociation of region I, while region II remains bound to the SNARE complex. This allows Synaptotagmin-1 to interact with PIP2-containing membranes and act as a lever that pulls the SNARE complex to facilitate membrane fusion [24,26]. The mechanism by which calcium binding to Syt1 translates into membrane fusion has been a long-standing question. The “lever hypothesis” offers an explanation: upon calcium binding, the Syt1 C2B domain undergoes a reorientation on the membrane [27]. This reorientation leads to a selective dissociation from SNAREs at region I of the primary interface while maintaining contact at region II. This differential interaction and conformational change allows Syt1 to act as a remote lever, exerting a pulling force on the SNARE complex [27]. This mechanical action facilitates the final zippering of SNARE helices required for rapid fusion. This Syt-1-mediated ‘lever’ mechanism primarily governs small synaptic vesicles (SVs). In contrast, large dense-core vesicles (LDCVs) transport neuropeptides and neuromodulators via distinct kinetics, exhibiting a heavier reliance on the high-affinity Ca^2+^ sensor Syt-7. Unlike SVs, DCV exocytosis displays unique redundancies in the membrane-binding domains of RIM and Munc13, reflecting a different evolutionary requirement for spatiotemporal precision compared to fast neurotransmission [4,21]. This lever model specifically accounts for synchronous release; in contrast, asynchronous release likely involves high-affinity sensors such as Syt7 or Doc2, which operate when the primary interface is not yet mechanically locked by Syt1 or through distinct Ca^2+^-independent reorientation pathways, reflecting the diverse calcium sensitivities observed in different release modes [24,27]. This explanation resolves a previous paradox where Syt1’s calcium-binding loops were oriented away from the fusion site, demonstrating a sophisticated biophysical mechanism for calcium-triggered release [27]. This mechanistic understanding of Synaptotagmin-1’s action as a calcium-activated lever represents a significant advancement, moving beyond simply identifying Syt1 as a calcium sensor to detailing the precise biophysical process by which it initiates membrane fusion. This indicates tight mechanical coupling at the synapse.

The assembly of the SNARE complex is itself orchestrated by other critical proteins (Table 1). Munc18-1 binds to synaptobrevin and to a “closed” conformation of syntaxin-1, effectively forming a template that guides SNARE complex assembly [22]. Munc13-1 further facilitates this assembly process by bridging the synaptic vesicle and plasma membranes. Following fusion, the SNARE complex is disassembled by the N-ethylmalemaleimide sensitive factor (NSF) and soluble NSF attachment proteins (SNAPs), allowing the SNARE proteins to be recycled for subsequent rounds of neurotransmitter release [22]. The interplay between these priming and fusion components underscores the highly coordinated molecular cascade that ensures synaptic vesicles are readily available and prepared for rapid release upon neuronal excitation. The observation that Munc18-1 and Munc13-1 orchestrate SNARE complex formation and vesicle priming reveals that neurotransmitter release is a multi-step process, not just a single fusion event [22]. This implies that any disruption in these priming components can directly impair release efficiency, even if the core fusion machinery remains intact [28].

**Table 1:** Key presynaptic active zone proteins and their roles in neurotransmitters release.

Protein	Role	Interactions
SNARE Complex (Syntaxin-1, SNAP25, Synaptobrevin)	Core machinery for membrane fusion; pulls vesicle and plasma membranes together	Synaptotagmin-1, Munc18-1, Munc13-1
Synaptotagmin-1 (Syt1)	Major Ca^2+^ sensor; triggers release via a “lever” mechanism	SNARE complex (primary interface regions I & II), membrane phospholipids
Munc13-1	Facilitates SNARE complex assembly; vesicle priming	RIM1, CAST/ELKS, Munc18-1
Rab3-Interacting Molecules (RIMs)	Active zone organization; synaptic vesicle docking and priming	Munc13-1, CAST/ELKS, Bassoon, Piccolo
Bassoon	Large scaffolding protein of the cytomatrix at the active zone (CAZ); AZ assembly, CaV localization, SV priming, ubiquitin-proteasome regulation	CAST/ELKS, RIMs, Piccolo
Piccolo	Large scaffolding protein of CAZ; AZ assembly, CaV localization, SV priming, actin cytoskeleton dynamics	CAST/ELKS, Bassoon
CAST/ELKS (Cytomatrix at Active Zone-Associated Structural Protein)	Key structural component of CAZ; links other AZ proteins	RIM1, Bassoon, Piccolo
Laminin β2	Postsynaptic synapse organizer; anchors presynaptic complex	Extracellular domains of presynaptic calcium channels
Voltage-gated Calcium Channels (CaV)	Mediate Ca^2+^ influx for release; presynaptic scaffolding	Cytosolic AZ proteins, Laminin β2

### 2.3. Synaptic Vesicle Recycling Pathways

To sustain the high rates of neurotransmission characteristic of neuronal activity, synaptic vesicles (SVs) undergo a highly efficient and continuous recycling process within the presynaptic terminal [28,29]. Following exocytosis, where SVs fuse with the presynaptic plasma membrane to release neurotransmitters, their components are retrieved from the membrane via various endocytic pathways and subsequently regenerated into new, fusion-competent SVs. This constant turnover ensures that neurotransmitter supply remains robust even during sustained high-frequency firing [29].

Several distinct endocytic pathways operate at the presynaptic terminal, each with characteristic kinetics and molecular machinery.

Clathrin-mediated endocytosis (CME) is the most prevalent pathway for SV retrieval. It involves the formation of clathrin-coated pits that bud off from the plasma membrane, internalizing SV components within tens of seconds following full-collapse fusion [30,31]. Key proteins involved include clathrin itself and dynamin, which mediates the scission of the budding vesicle from the plasma membrane [32]. CME typically occurs in the peri-active zone, adjacent to the sites of neurotransmitter release [12,28].

Kiss-and-run is a rapid mechanism that involves a transient opening of a fusion pore, allowing for neurotransmitter release without full collapse of the SV into the plasma membrane [30]. The vesicle is then rapidly retrieved, often within approximately one second [31,32]. This pathway allows for very fast recycling, particularly important during moderate activity.

Bulk endocytosis is triggered by intense or prolonged neuronal stimulation. This pathway involves the retrieval of large portions of the plasma membrane in the form of large endocytic vacuoles [30]. New SVs are subsequently regenerated from these larger structures through clathrin-mediated budding [12,31].

Ultrafast endocytosis occurs rapidly, within 50–100 milliseconds following stimulation, capturing approximately four vesicles at the very edge of the active zone [32]. This mechanism is specialized for rapidly restoring the surface area of the presynaptic membrane and is kinetically distinct from both kiss-and-run and clathrin-mediated endocytosis [33].

Calcium ions play a fundamental regulatory role in orchestrating these diverse endocytic processes across different synapse types [12]. The existence of multiple endocytic pathways suggests an adaptive recycling system. The molecular switch shifting retrieval toward ultrafast endocytosis is primarily mechanical, driven by a ‘membrane compression model’ where fused synaptic vesicles exert lateral pressure against an F-actin-enriched stiff periactive zone, inducing rapid membrane buckling and curvature independent of clathrin recruitment [31,34]. This system is capable of adjusting their retrieval mechanisms based on the intensity and pattern of neuronal activity. The observation that ultrafast endocytosis is triggered by strong stimulation and operates significantly faster than clathrin-mediated endocytosis indicates coordinated regulation that matches recycling kinetics to the demands of sustained neurotransmission [32]. This adaptability is crucial for preventing the depletion of SVs and avoiding excessive expansion of the plasma membrane, thereby maintaining synaptic efficiency and integrity [23].

Despite significant progress in identifying many of the proteins involved, a complete and unified understanding of how SV recycling is controlled as a whole remains elusive [23]. Key questions persist regarding whether a single, overarching model for SV recycling exists, or if specialized mechanisms are uniquely adapted for particular synapse types or specific physiological conditions [35]. The precise mechanisms that govern the number of vesicles undergoing exo- or endocytosis, ensuring the efficient allocation of cellular resources, are still subjects of active investigation [23]. A significant controversy persists regarding the relative contributions of these pathways, as clathrin-mediated endocytosis is predominantly observed in in vitro models using non-physiological temperatures or intense stimulation [12]. In contrast, ultrafast endocytosis appears to be the primary mechanism under physiological temperatures and in vivo conditions, suggesting that many classical observations of coated pits may be artifacts of chemical fixation or thermal stress [12]. Furthermore, differences in vesicle mobilization between dissociated cultures and organotypic slices raise questions about the generalizability of certain in vitro recycling models to the intact brain [12,13,33]. Further research is needed to integrate these diverse pathways into a comprehensive, systems-level model that fully explains the dynamic control of synaptic vesicle homeostasis.

## 3. Postsynaptic Molecular Mechanisms

### 3.1. Postsynaptic Density (PSD) Structure and Protein Composition

The postsynaptic density (PSD) is a highly organized, electron-dense protein complex situated directly beneath the postsynaptic membrane of excitatory synapses [36]. This structure, typically found at the tip of dendritic spines, serves as a crucial hub for signal reception, transduction, and synaptic plasticity. The PSD’s dynamic nature and precise molecular organization are essential for modulating synaptic strength and ensuring efficient neuronal communication [36].

The PSD is characterized by high molecular complexity, comprising over a thousand different proteins in mammals [9]. This diverse proteome includes a wide array of neurotransmitter receptors, scaffolding proteins, signaling enzymes, cytoskeletal components, and cell adhesion molecules. Cryo-electron tomography studies have revealed that the PSD is not a monolithic structure but is composed of clustered, heterogeneous nanoblocks that vary in size, assembly, and distribution [36]. Nanoblocks contains two specific types of receptor-like particles, type A (O-shaped) and type B (Y-shaped) [36]. Presynaptic release sites align preferentially with nanoblocks containing these A/B particles, while nanoblocks lacking them may serve as spare scaffolds or house other receptors [36]. This nanoscale organization contributes significantly to the dynamic properties of the PSD. During a single LTP event, this heterogeneity is resolved through rapid structural remodeling where ‘nascent zones’ are converted into functional active zones within 5 min via the recruitment of dense-core vesicles, representing a high turnover rate of these nanoblocks to saturate potentiation capacity [36,37]. The multi-layered hierarchical organization of the PSD, with its distinct core and pallium layers and heterogeneous nanoblocks, suggests that it is an adaptable structure. This modularity implies that different components can be independently regulated or interact in a coordinated manner to fine-tune synaptic strength and plasticity, allowing for precise control over receptor localization and signaling [36].

Beyond protein–protein scaffold interactions, emerging evidence implicates cytoskeletal elements in PSD structural maintenance. Recent in vitro work suggests that the PSD may be dynamically maintained not only by canonical scaffold assemblies, but also by interactions between a tubulin-containing PSD lattice/backbone and polymerizing microtubules. Using electron microscopy, Suzuki et al. reported physical associations between postsynaptic structures and growing microtubules, consistent with a model in which transient microtubule intrusions into spine heads can couple to PSD structural remodeling. Notably, the authors found that exposing PSD/PSD-lattice preparations to GTP and microtubule-affecting reagents compromises PSD structural integrity, and that GTP can drive distinct outcomes depending on whether polymerizing tubulin is present, ranging from structural disassembly in the absence of polymerizing tubulin to enlargement/reorganization when polymerizing microtubules are available. Together, these findings support a view of the PSD as an inherently dynamic structure that balances disassembly and reconstruction, with the tubulin-microtubule system and GTP availability acting as modulators of PSD maintenance and reorganization [38].

Key scaffolding proteins form the structural backbone of the PSD. PSD-95, a prominent member of the MAGUK family, is a major structural component of the PSD core layer [39]. It is oriented vertically to the postsynaptic membrane, strategically positioned to bind and organize both AMPA and NMDA glutamate receptors [40,41]. PSD-95, along with other related MAGUK molecules, forms a dense matrix that provides a stable platform for the entire postsynaptic apparatus. This platform is further supported by interactions with proteins like GKAP (guanylate kinase-associated protein), which forms an interface between PSD-95 and deeper PSD layers [39].

Deeper within the PSD, in a region often referred to as the “pallium,” Shank and Homer proteins self-assemble into a mesh-like matrix characterized by tetrameric coiled-coils [42]. Immunogold electron microscopy places Homer in a layer 30–100 nm from the postsynaptic membrane, whereas mGluRs are concentrated at peri-PSD locations within 60 nm of the edge [43]. This organization serves as a structural framework and an assembly platform for other PSD proteins, including GKAP [42]. The dynamic addition of proteins like CaMKII to the pallium during periods of intense synaptic activity further demonstrates the PSD’s adaptability, influencing spine morphology and receptor trafficking [39].

The integrity and proper function of PSD proteins are paramount, as abnormalities have been strongly linked to various neuropsychiatric diseases, including autism spectrum disorder and schizophrenia [9]. The PSD may be vulnerable due to its central role in integrating signals and organizing synaptic transmission and plasticity [10]. Disruptions in the hierarchical assembly of proteins within the “synaptome architecture” can lead to widespread behavioral phenotypes, highlighting the PSD as a critical target for understanding and treating complex neurological and psychiatric conditions [10].

### 3.2. Excitatory Receptor Trafficking and Regulation

Fast excitatory synaptic transmission in the central nervous system is mediated by two classes of ionotropic glutamate receptors, AMPA receptors (AMPARs) and NMDA receptors (NMDARs) and metabotropic glutamate receptors (mGluRs). Group I mGluRs are regulated by surface expression and trafficking through interactions with specialized PSD proteins. Maladaptive functioning of these PSD scaffold proteins is as critical as mGluR dysfunction itself in the pathophysiology of various neuropsychiatric disorders, offering novel targets for therapeutics [44]. The precise number, subtype, and localization of glutamate receptors at the postsynaptic membrane are dynamically regulated through complex trafficking mechanisms, which are crucial for synaptic plasticity and cognitive functions like learning and memory [45].

#### 3.2.1. AMPAR Trafficking and Regulation

**Figure 2:**
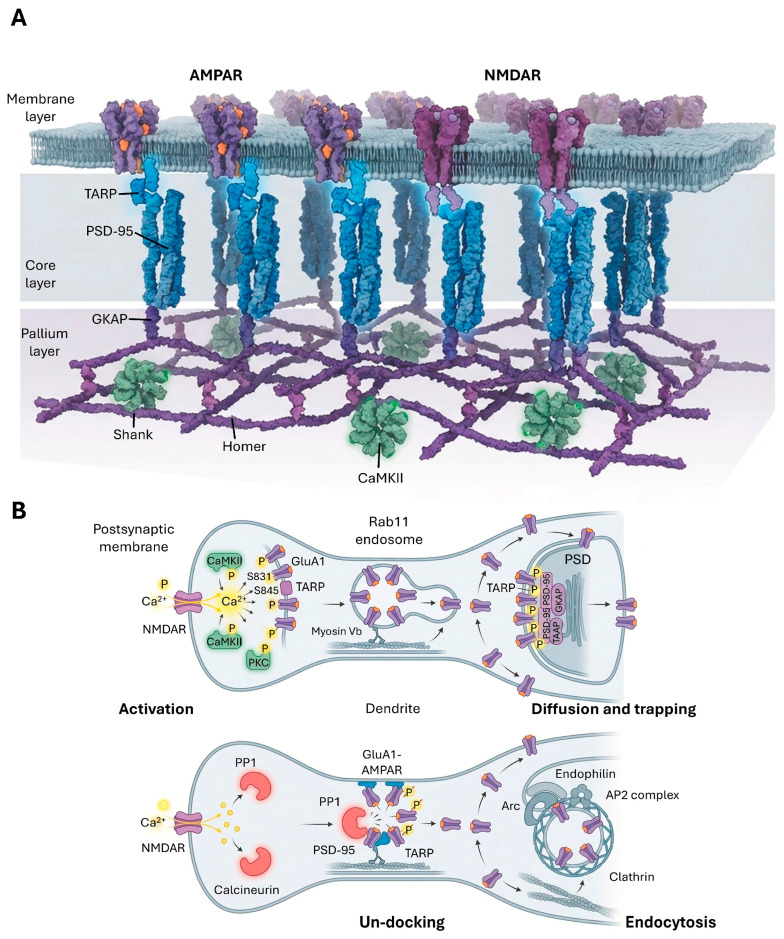
Hierarchy of the postsynaptic density and AMPAR trafficking. (**A**) Layered organization of the postsynaptic density (PSD) comprising the membrane layer (receptors and TARPs), the core layer (PSD-95), and the pallium layer (Shank and Homer). (**B**) Molecular pathways of AMPA receptor regulation. LTP induction (top) involves CaMKII-mediated phosphorylation (S831) and Myosin Vb-driven exocytosis from Rab11 endosomes. LTD (bottom) is characterized by PP1/calcineurin-mediated dephosphorylation and Arc-dependent clathrin-mediated endocytosis.

AMPARs are tetrameric proteins, typically composed of GluA1, GluA2, GluA3, and GluA4 subunits, with heterotetrameric combinations (e.g., GluA1/GluA2 or GluA2/GluA3) being common in the adult brain [45]. Their continuous trafficking to and from the synaptic surface, occurring within seconds to minutes, is a critical determinant of synaptic efficacy and underlies both long-term potentiation (LTP) and long-term depression (LTD) [45] (Figure 2).

During LTP induction, GluA1-containing AMPARs are rapidly incorporated into the synaptic surface of excitatory synapses. These receptors then undergo lateral diffusion through the membrane to enter the postsynaptic density (PSD), where their retention is facilitated by interactions with PSD-95 via transmembrane AMPA receptor regulatory proteins (TARPs) [45]. This process is tightly regulated by phosphorylation events. Thus, CaMKII, activated by calcium influx during LTP, phosphorylates GluA1 at the S831 site and TARPs at S277 and S281 sites. This phosphorylation enhances AMPAR single-channel conductance and promotes their trapping at the synaptic surface, leading to synaptic strengthening [45]. Additionally, synaptic transmission and plasticity are fundamentally tuned by GPCR-mediated neuromodulation. Synaptic transmission and plasticity are fundamentally tuned by GPCR-mediated neuromodulation. The Gs/cAMP/PKA cascade, often triggered by adhesion-GPCRs like latrophilins, is essential for initial AMPAR surface recruitment by facilitating the insertion of receptor packets, whereas Gq/PLC/PKC signaling events, modulated by group I metabotropic glutamate receptors (mGluR1/5), orchestrate receptor exocytosis and structural remodeling [1]. Furthermore, Gi/o-mediated pathways, such as those activated by the purinergic P2Y12 receptor, provide a critical break on release probability and coordinate microglial-synapse interactions during circuit refinement [1,12,45,46].

The exocytosis of GluA1-containing AMPARs to the perisynaptic surface involves the motor protein Myosin Vb, which transports endosomes containing these receptors via Rab11 and its effector Rab11-FIP2 [45]. The final membrane fusion step is mediated by calcium-sensor synaptic vesicle protein Syt1, along with Syt7, Synaptobrevin 2 (Syb2/VAMP2), and complexin. Scaffold proteins also play crucial roles. Protein 4.1N, abundant in dendritic spines, interacts with SynCAM1 and phosphorylated GluA1 (S818) to facilitate AMPAR recruitment and exocytosis [45]. Synapse-associated protein 97 (SAP97), another MAGUK family protein, acts as an adaptor between GluA1 and Myosin V, being essential for intracellular trafficking and targeting of AMPARs during LTP [47]. The subunit-specific and activity-dependent regulation of AMPAR trafficking is critical for synaptic strength and plasticity. The detailed mechanisms involving different phosphorylation sites and interacting proteins (CaMKII, PKA, PKC, Myosin Vb, Protein 4.1N, SAP97, Arc, protein interacting with C kinase 1 (PICK1), adaptor protein-2 (AP2) complex) during exocytosis, lateral diffusion, and endocytosis highlight that the neuron precisely controls synaptic strength by fine-tuning the composition and number of AMPARs at the postsynapse through a complex, multi-faceted regulatory network [45].

Conversely, LTD is characterized by the removal of GluA1-containing AMPARs from the synaptic surface. This process involves the dissociation of GluA1 subunits from the GluA1-TARP-(PSD-95) complex, followed by dephosphorylation of TARP by protein phosphatase 1 (PP1) and dephosphorylation of GluA1 at the S845 site, which collectively promote receptor endocytosis [45]. GluA2 subunits are also internalized during LTD, a process involving PKCα-mediated phosphorylation at the S880 site, which disrupts their binding to GRIP1. The liberated GluA2 subunits then bind to PICK1, diffuse to the endocytic zone, and undergo clathrin-mediated endocytosis facilitated by interaction with the AP2 complex [45]. Internalized receptors can either be recycled back to the surface during subsequent LTP or degraded in lysosomes during LTD. Arc (activity-regulated cytoskeleton-associated protein) plays a critical role in AMPAR endocytosis during LTD and homeostatic scaling down by having a higher affinity for dephosphorylated TARP and facilitating endocytosis via AP2 and endophilin [45].

#### 3.2.2. NMDAR Trafficking and Regulation

**Figure 3:**
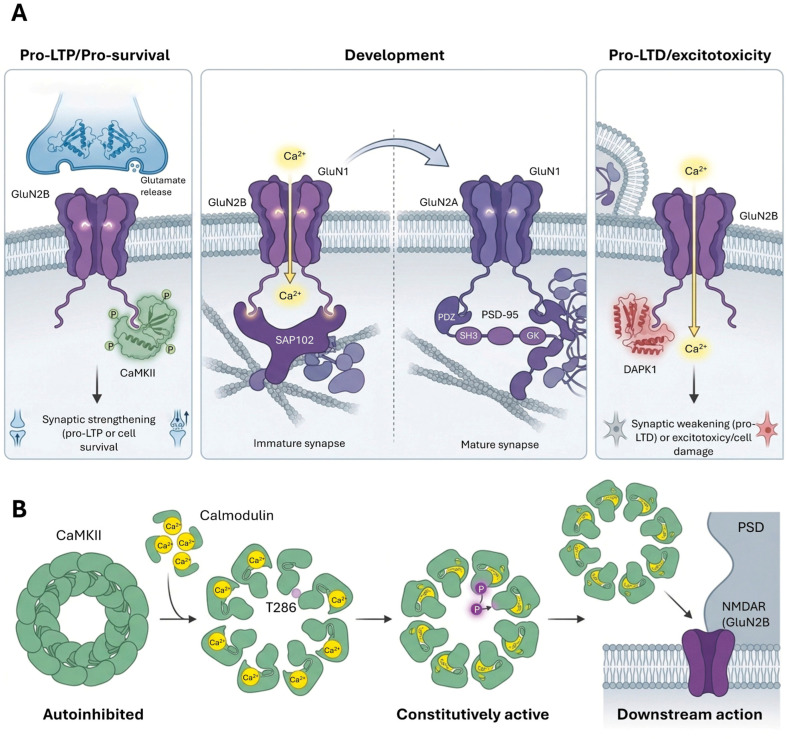
NMDAR subunit composition and CaMKII activation. (**A**) Developmental and functional diversity of NMDA receptors. Immature synapses predominantly express GluN2B (linked to SAP102), while mature synapses shift toward GluN2A (linked to PSD-95). Extrasynaptic GluN2B activation by DAPK1 is associated with excitotoxicity. (**B**) The biochemical switch of CaMKII. Following Ca^2+^/calmodulin binding, T286 autophosphorylation renders the kinase constitutively active, allowing it to translocate to the PSD and bind the GluN2B subunit to initiate downstream plasticity events.

NMDARs are crucial for synaptic plasticity and memory formation, functioning as “coincidence detectors” that require both presynaptic glutamate release and postsynaptic depolarization to open their ion channel and allow for calcium influx [48]. Functional NMDARs are heterotetramers composed of two obligatory GluN1 subunits and two GluN2 (GluN2A-D) or GluN3 subunits. The specific GluN2 subunits confer distinct ion channel properties and intracellular trafficking pathways, and their expression patterns vary developmentally and spatially across the central nervous system [48] (Figure 3).

A significant developmental switch occurs in higher brain regions, transitioning from predominantly GluN2B-containing NMDARs in immature synapses to GluN2A-containing NMDARs in mature synapses [40]. This subunit switch profoundly alters NMDAR-mediated calcium influx, thereby impacting dendritic integration and synaptic plasticity rules [48]. This activity-dependent switch is mediated by metabotropic glutamate receptor (mGluR) signaling, intracellular calcium release, and the activation of protein kinase C (PKC) and phospholipase C (PLC), with casein kinase 2 (CK2) phosphorylation of GluN2B promoting its internalization. The distinct biophysical properties and spatiotemporal expression patterns of different GluN2 subunits emphasize that NMDARs are not a homogeneous group [48]. The developmental shift from GluN2B to GluN2A signifies a fundamental change in synaptic function and plasticity, implying that therapeutic interventions targeting NMDARs must consider subunit specificity to avoid unintended effects.

NMDAR trafficking is a complex process involving biosynthesis, dendritic transport, exocytosis, lateral diffusion, endocytosis, recycling, and degradation, all tightly controlled by protein–protein interactions involving the cytoplasmic carboxyl termini (CTDs) of NMDAR subunits [48]. MAGUK proteins, including PSD-95 and synapse-associated protein 102 (SAP102), are primary scaffolds that interact with GluN2 subunits, anchoring and stabilizing NMDARs at glutamatergic synapses [40]. Notably, SAP102 mediates the synaptic trafficking of both AMPA and NMDA receptors during early synaptogenesis [49], while PSD-95 assumes this function during synapse maturation, including the crucial replacement of NR2B-NMDARs with NR2A-NMDARs [40,50].

NMDARs exhibit lateral diffusion on the plasma membrane, moving between synaptic and extrasynaptic sites [48]. GluN2B-containing NMDARs are generally more mobile than GluN2A-containing receptors, which tend to be more stably localized at synapses. Synaptic retention of GluN2A requires its CTD for stable interactions with PDZ domain-containing scaffolding molecules like PSD-95.16 NMDAR endocytosis is primarily clathrin-mediated and involves the adaptor protein-2 (AP-2) complex [48]. GluN2B-containing receptors show higher internalization rates in mature neurons, with their internalization modulated by phosphorylation of Tyr-1472 by Fyn kinase. Sorting Nexin 27 (SNX27) is also implicated in regulating NMDAR recycling from endosomes back to the plasma membrane [48,50]. However, super-resolution mapping using DNA-PAINT has revealed that NMDARs are typically absent from the region immediately opposing Munc13-1 release sites. Only a subset of release sites aligned with PSD-95 forms enriched ‘nanocolumns’ containing GluN2A/B, where NMDAR activation rapidly reorganizes this topography to tune synaptic transmission [50]. This spatial segregation allows independent release sites to activate unique ratios of NMDAR subunits, expanding the computational capacity of single synapses [50].

CaMKII, a highly abundant protein enriched in the PSD, is critically involved in NMDAR-dependent LTP [51,52]. Upon NMDAR activation and calcium influx, CaMKII becomes activated and translocates to the PSD, where it binds to GluN2B. This interaction initiates structural rearrangements within the PSD and promotes the accumulation of AMPARs, contributing to synaptic potentiation [53]. CaMKII also phosphorylates GluN2B at Ser-1303, which can reduce its own binding and increase surface GluN2B expression [48].

In the context of excitotoxicity, particularly relevant in neurological disorders, death-associated protein kinase 1 (DAPK1) is recruited to extrasynaptic GluN2B-containing NMDARs [48]. Activated DAPK1 phosphorylates GluN2B at Ser-1303, enhancing channel conductance and leading to detrimental calcium influx.16 DAPK1 also plays a role in LTD, where its activation via calcineurin-dependent dephosphorylation leads to GluN2B phosphorylation at Ser-1303, strengthening DAPK1 interaction and preventing CaMKII binding. This highlights the differential roles of NMDAR subtypes and their localization in neuronal health and disease. Beyond its pathological role in excitotoxicity, DAPK1 functions as a refined physiological regulator during long-term depression (LTD), where its recruitment to GluN2B prevents CaMKII accumulation in the spine, thereby enabling bidirectional control of synaptic strength [48,54].

### 3.3. Inhibitory Receptor Trafficking and Regulation

Inhibitory synaptic transmission is crucial for balancing neuronal excitability and shaping neural circuit activity. The primary inhibitory neurotransmitter in the central nervous system is gamma-aminobutyric acid (GABA), which is synthesized from the excitatory neurotransmitter glutamate [55]. GABA exerts its effects through GABAA, GABA-B, and GABA-C receptors, and maintaining its balance with glutamate is essential for cell membrane stability and proper neurological function gephyrin/CB lattice [55]. Glycine receptors (GlyRs) also mediate synaptic inhibition, particularly in the brainstem and spinal cord [56]. The precise localization and stability of these inhibitory receptors at postsynaptic sites are mediated by specialized scaffolding proteins.

#### 3.3.1. GABA-A Receptor Trafficking and Regulation

**Figure 4:**
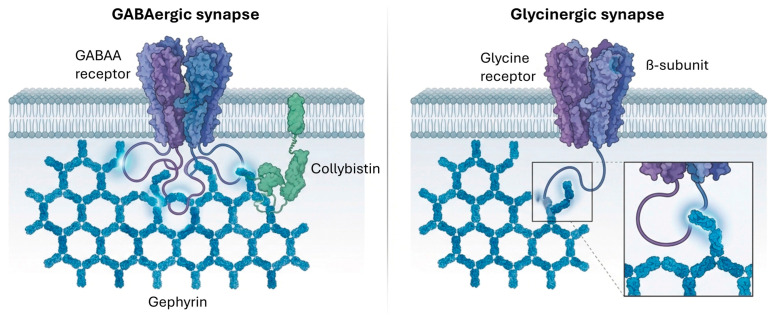
Scaffolding mechanisms at inhibitory synapses. Molecular organization of GABAergic and glycinergic postsynaptic sites. Gephyrin forms a planar lattice that anchors GABA-A receptors and Glycine receptors via their intracellular loops. Collybistin acts as a GEF to anchor the gephyrin lattice to the membrane, ensuring the stability of inhibitory transmission.

GABA-A receptors (GABAARs) are ligand-gated ion channels that typically exist as heteropentamers composed of various combinations of α, β, γ, ρ, δ, ε, π, and θ subunits [55]. The subunit composition dictates their biophysical properties and subcellular localization, determining whether they mediate phasic (synaptic) or tonic (extrasynaptic) inhibition. For instance, the γ2 subunit is essential for synaptic localization, while δ subunit-containing GABAARs are typically extrasynaptic [55] (Figure 4).

Gephyrin is a multifunctional scaffold protein that forms submembranous postsynaptic planar lattices at both GABAergic and glycinergic synapses [57]. Gephyrin is essential for the clustering and anchoring of a large proportion of synaptic GABAARs, though not all. The postsynaptic scaffold also includes collybistin (CB), which binds to gephyrin and membrane phosphoinositides, thereby anchoring the gephyrin/CB lattice to the postsynaptic membrane [57]. Collybistin acts as a guanine nucleotide exchange factor (GEF) for the small GTPase Cdc42, and its deficiency leads to disrupted postsynaptic clustering of GABAARs and Gephyrin in various brain regions, including the hippocampus, amygdala, and cerebellum [57].

GABAARs are initially delivered to the cell surface at non-synaptic sites via exocytosis and then undergo lateral diffusion in the plasma membrane to be trapped at the synapse. This trapping is thought to occur through the interaction of the large intracellular loop (IL) of the α subunits of synaptic GABAARs with the postsynaptic Gephyrin/CB lattice. Studies have shown that the ILs of α1, α2, α3, or α5 subunits bind to monomeric gephyrin in vitro and to collybistin, playing a critical role in their synaptic localization [57]. Notably, the IL of the α2 GABAAR subunit not only binds to gephyrin but also activates collybistin, which is important for targeting α2 GABAARs to the axon initial segment. The β3 subunit appears to be a major determinant for the interaction of GABAARs with gephyrin/CB clusters [57].

Experimental reconstitution studies in HEK293 cells have demonstrated that the gephyrin/CB lattice can directly trap various GABAAR subunit compositions, including typically synaptic α1β3γ2 GABAARs and some extrasynaptic subtypes like α6β3δ and α1β3. However, other extrasynaptic receptors, such as α4β3δ, show little or no trapping, suggesting that additional neuronal mechanisms are at play to prevent the trapping of certain extrasynaptic GABAARs by the gephyrin/CB lattice in neurons [57]. This indicates that while the gephyrin/CB scaffold is crucial, it is not the sole determinant for GABAAR synaptic localization, and other synaptic molecules (e.g., GARLH4, neuroligin 2) and post-translational modifications (e.g., phosphorylation of gephyrin and GABAAR subunit ILs) also contribute to the complex regulation of GABAAR clustering and synaptic localization [57]. Gephyrin post-translational modifications, particularly phosphorylation, regulate the recruitment of the prolyl-isomerase Pin1. This mechanism is essential for the structural remodeling of GABAergic synapses. Specifically, gephyrin phosphorylation within its central C-domain recruits the prolyl-isomerase Pin1, triggering conformational changes that modulate the gephyrin lattice’s oligomerization. Specifically, the C3 splice cassette strongly impacts E-domain stability, resulting in a reduction in the melting temperature by nearly 12 °C, which favors a more homogeneous cytosolic distribution over membrane-anchored lattices [58]. These modulate its affinity for GABAergic and glycinergic receptors, thus balancing inhibitory stability with plastic remodeling [57,58,59,60]. Genetic mutations or alterations in gephyrin expression are implicated in several disorders, specifically including temporal lobe epilepsy, as well as autism and Alzheimer’s disease [59].

#### 3.3.2. Glycine Receptor Trafficking and Regulation

Glycine receptors (GlyRs) are ligand-gated chloride channels that mediate fast inhibitory neurotransmission, predominantly in the spinal cord and brainstem, but also play roles in embryonic brain development, learning, memory, and pain sensitization. 71 GlyRs are typically composed of α and β subunits, which assemble into homomeric α-pentamers or α2β3 heteromeric receptors [61].

The synaptic localization of GlyRs is critically dependent on their interaction with the postsynaptic scaffolding protein gephyrin [58]. Gephyrin binds with high affinity to the large cytoplasmic loop of the GlyR β-subunit (GlyRβ), anchoring the receptor to the subsynaptic cytoskeleton and forming postsynaptic clusters [56]. Beyond gephyrin, other proteins are also involved in GlyR trafficking and anchoring.

Syndapin I (SdpI) has been identified as a novel interaction partner of GlyRβ, co-immunoprecipitating with native GlyRs from brainstem extracts [61]. Both Syndapin I and Syndapin II bind efficiently to the intracellular loop of GlyRβ in vitro and co-localize with GlyRβ upon co-expression in cells, suggesting a role in GlyR trafficking to and/or anchoring at inhibitory postsynapses. Additionally, trafficking proteins such as vacuolar protein sorting 35 (Vps35) and neurobeachin (Nbea) have been found to interact with GlyRβ, involving loop residues adjacent to the gephyrin-binding motif [61].

Despite these advancements, the full complement of proteins interacting with native GlyRs remains largely unknown, with fewer than 20 potential interactors identified to date compared to other neurotransmitter receptors [62]. Many known interactors either bind specifically to the GlyR α1 and β subunits, or their precise binding partner within the GlyR complex is unclear. Furthermore, some identified GlyR interactors are not found at inhibitory synapses or lack a clear functional role, while others are secondary interactors that bind indirectly, for example, via gephyrin [62]. Understanding the identity and roles of GlyR accessory proteins is vital for deciphering GlyR function and dysfunction in health and disease, particularly given the emergence of disease-associated missense mutations in the α1, α2, and β subunit intracellular domains linked to startle disease (associated with GlyR α1 and β subunits) and neurodevelopmental disorders such as autism, intellectual disability, and epilepsy, which are specifically linked to the GlyR α2 subunit [62].

## 4. Synaptic Plasticity: Molecular Mechanisms

Synaptic plasticity, the ability of synapses to strengthen or weaken over time in response to activity, is the cellular basis for learning and memory in the brain. This dynamic modulation of synaptic strength involves molecular cascades that lead to both functional and structural changes at the synapse. The two most extensively studied forms of synaptic plasticity are long-term potentiation (LTP) and long-term depression (LTD).

### 4.1. Long-Term Potentiation (LTP)

Long-term potentiation (LTP) is a persistent increase in synaptic strength induced by brief periods of high-frequency presynaptic stimulation. This process is crucial for long-term memory formation and can last for extended periods, from hours to months in vivo.

The induction of LTP at many central synapses, particularly in the hippocampus, is primarily dependent on the activation of NMDARs [53]. NMDARs function as “coincidence detectors,” requiring both presynaptic glutamate release and sufficient postsynaptic depolarization to relieve their magnesium block and allow for calcium influx into the dendritic spine. This calcium influx is the critical trigger for LTP. Emerging evidence suggests a major conceptual shift: the role of CaMKII in LTP maintenance is driven largely by structural rearrangements rather than its enzymatic activity alone [63]. Upon activation, CaMKII acts as a persistent structural ‘seed’ or molecular tag that captures proteins, such as densin and delta-catenin, required for spine growth and synaptic stabilization. This complex maintains the ‘on-state’ of synaptic memory through physical interactions within the PSD, effectively functioning as a structural switch that resists protein turnover [51,52,63,64]. However, evidence indicates that LTP is compound in nature, involving both increased presynaptic release probability and enhanced postsynaptic conductance [65]. Furthermore, enriched learning modes elevate intracellular calcium levels, promoting CaMKII-mediated cytoskeletal remodeling to strengthen stored memories [66].

A key downstream effector of this calcium signal is calcium/calmodulin-dependent protein kinase II (CaMKII). CaMKII is highly abundant in the postsynaptic density (PSD) and is rapidly activated by calcium/calmodulin binding following NMDAR activation [52]. Upon activation, CaMKII undergoes autophosphorylation at Thr-286, which renders it constitutively active even after calcium levels return to baseline. Activated CaMKII then translocates to the PSD, where it binds to the cytoplasmic C-tail of the GluN2B subunit of NMDARs. This interaction is crucial for LTP induction and is thought to initiate structural rearrangements within the PSD [52].

The primary mechanism by which CaMKII mediates early LTP is through the phosphorylation of AMPARs) and their auxiliary subunits, such as TARPs. Specifically, CaMKII phosphorylates the GluA1 subunit of AMPARs at the S831 site, which increases AMPAR single-channel conductance and promotes their trapping at the synaptic surface, leading to synaptic strengthening [45]. CaMKII also phosphorylates TARPs, enhancing the retention of GluA1-containing AMPARs within the PSD by forming a GluA1-TARP-(PSD-95) complex. Other kinases, such as protein kinase A (PKA) and protein kinase C (PKC), also contribute to AMPAR trafficking during LTP. Synaptic transmission and plasticity are fundamentally tuned by GPCR-mediated neuromodulation. The Gs/cAMP/PKA cascade, often triggered by adhesion-GPCRs like latrophilins, is essential for initial AMPAR surface recruitment, whereas Gq/PLC/PKC signaling events—modulated by group I metabotropic glutamate receptors (mGluR1/5)—orchestrate receptor exocytosis and structural remodeling [5,67]. Furthermore, Gi/o-mediated pathways provide a critical break on release probability, illustrating how diverse G-protein coupling provides temporal and gain control over synaptic output [4,45].

Beyond these functional changes, CaMKII also plays a role in the later stages of LTP by contributing to structural changes in the synapse, including the remodeling of the actin cytoskeleton within dendritic spines [52]. Astrocytes participates in LTP supplying glycine [68].

### 4.2. Long-Term Depression (LTD)

Long-term depression (LTD) is a persistent decrease in synaptic strength, often induced by prolonged low-frequency stimulation or specific patterns of activity. Like LTP, LTD is a fundamental mechanism underlying learning and memory, providing a means for synaptic weakening and circuit refinement. Similar to LTP, LTD is also calcium-dependent, but typically involves a smaller or more prolonged calcium influx compared to LTP. This differential calcium signal activates protein phosphatases, such as protein phosphatase 1 (PP1) and calcineurin (protein phosphatase 2B), which dephosphorylate key synaptic proteins [45].

A primary mechanism of LTD involves the removal of AMPA receptors from the postsynaptic membrane. This process is initiated by the dephosphorylation of TARPs by PP1, which disrupts their interaction with PSD-95 and releases GluA1 subunits.15 Subsequent dephosphorylation of GluA1 at the S845 site further promotes receptor endocytosis.15 GluA2 subunits are also internalized during LTD, a process that involves PKCα-mediated phosphorylation at the S880 site, disrupting their binding to GRIP1. The liberated GluA2 subunits then bind to PICK1 and undergo clathrin-mediated endocytosis, facilitated by interaction with the AP2 complex. The Arc protein is a critical regulator of AMPAR endocytosis during LTD and homeostatic scaling down, by having a higher affinity for dephosphorylated TARP and facilitating the internalization of the GluA1-TARP-Arc complex via AP2 and endophilin [45].

LTD can be induced by both NMDAR-dependent and metabotropic glutamate receptor (mGluR)-dependent mechanisms. Interestingly, recent research indicates that NMDAR-dependent LTD also requires CaMKII and its autonomous (calcium-independent) activity. In this context, LTD stimuli induce CaMKII-dependent phosphorylation of GluA1 at S567, a site that reduces synaptic localization and is unaffected by Ca^2+^/CaM stimulation, unlike the S831 site favored in LTP [69]. This enables autonomous CaMKII to mediate opposing forms of plasticity through differential substrate site selection [69]. Furthermore, DAPK1 is implicated in LTD, where its activation via calcineurin-dependent dephosphorylation leads to GluN2B phosphorylation at Ser-1303, strengthening DAPK1 interaction and preventing CaMKII binding, contributing to synaptic weakening [45,48].

### 4.3. Structural Plasticity and Cytoskeletal Remodeling

**Figure 5:**
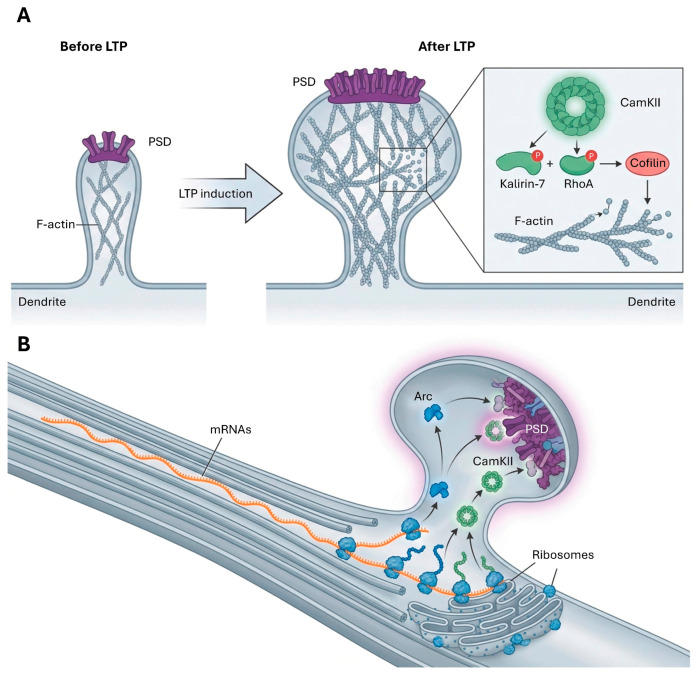
Structural plasticity and dendritic local translation. (**A**) Remodeling of the dendritic spine during LTP. CaMKII activation triggers actin polymerization through Kalirin-7 (Rac1) and RhoA while inactivating cofilin to expand the F-actin cytoskeleton. (**B**) Local protein synthesis at the synapse. mRNAs and ribosomes are localized near the spine to allow for activity-dependent translation of proteins like Arc, which are essential for long-term synaptic maintenance.

Beyond changes in receptor number and function, synaptic plasticity also involves structural remodeling of dendritic spines, the small protrusions on dendrites that receive most excitatory synaptic input. These morphological changes are crucial for the long-term maintenance of synaptic strength and memory storage (Figure 5).

During LTP, dendritic spines can enlarge and change shape, a process driven by the reorganization of the actin cytoskeleton within the spine [62]. CaMKII plays a central role in this process; upon activation, it dissociates from filamentous actin (F-actin), allowing the cytoskeleton to reorganize. CaMKII also phosphorylates key regulators of actin dynamics, such as kalirin-7 (which activates Rac1) and RhoA, leading to the functional inactivation of cofilin (an F-actin severing protein) and the addition of actin monomers to growing filaments, respectively. This coordinated action modifies the equilibrium between actin construction and destruction, facilitating spine expansion [62].

Conversely, LTD can lead to spine shrinkage or elimination. Three-dimensional reconstructions from electron microscopy reveal that during LTP induction, dense-core vesicles (DCVs) that carry tethered presynaptic vesicles are recruited to silent nascent zones (NZs). These DCVs dock and convert NZs into functional active zones (AZs) in less than 5 min, a process that saturates potentiation capacity until new NZs are generated [37]. During recovery, new NZs form on spines where AZs were most enlarged; these ‘sentinel spines’ are often associated with the presence of smooth endoplasmic reticulum (SER) and form clusters with smaller spines [37]. Conversely, LTD is associated with the loss of weak AZs, driving synapse shrinkage. However, neighboring synapses displaying coordinated activity act as mutual safeguards against pruning, ensuring that LTD-dependent elimination primarily impacts synapses that do not contribute to network activity responses [70]. This interplay between functional and structural changes ensures that synaptic strength is not only modulated but also physically maintained over time [62].

### 4.4. Role of Local Protein Synthesis

While early phases of LTP (E-LTP) are mediated by post-translational modifications in existing proteins and occur independently of protein synthesis, the long-lasting forms of synaptic plasticity, including late-phase LTP (L-LTP) and LTD, require new local protein synthesis within dendrites, a process where specificity is determined by RNA-binding proteins and activity-dependent capture [71,72]. This local translation of messenger RNAs (mRNAs) at or near synapses is a critical mechanism for maintaining persistent changes in synaptic strength and for memory consolidation (Figure 5).

Both LTP and LTD rely on similar signal transduction cascades that regulate translation initiation. For instance, activation of group 1 mGluRs induces LTD that requires rapid protein synthesis within minutes. Newly synthesized proteins are thought to maintain LTP and LTD primarily through the regulation of ionotropic glutamate receptor trafficking or the stabilization of structural changes that maintain receptor density at the synapse [17].

The specificity by which new proteins participate in either LTP or LTD may be determined by specific RNA-binding proteins and an “activity-dependent capture” mechanism. This suggests that while translation machinery may be broadly activated, the precise proteins synthesized and utilized are selectively integrated into the activated synapses. This activity-dependent capture involves a binding cascade of densin, delta-catenin, and N-cadherin, where the CaMKII/NMDAR complex serves as the initial molecular tag that captures these proteins to increase synapse size and stability [17,63]. Key signaling molecules like extracellular signal-regulated kinase (ERK) are involved in regulating translation initiation in both LTP and LTD paradigms.

## 5. Synapse Formation, Maturation, and Elimination

The formation, maturation, and selective elimination of synapses are dynamic processes essential for the precise wiring of neural circuits during development and for maintaining brain function throughout life [1,3]. These processes are guided by a complex interplay of molecular cues, cell adhesion molecules, and the activity of glial cells.

### 5.1. Molecular Cues and Cell Adhesion Molecules in Synaptogenesis

Synaptogenesis, the formation of new synapses, is initiated when axonal growth cones, extending from developing neurons, encounter their target cells and establish adhesive interactions [49,73,74]. This initial contact triggers a cascade of intracellular signaling events that lead to the recruitment and organization of presynaptic and postsynaptic components.

Cell adhesion molecules (CAMs) are pivotal in coordinating multiple steps of synaptogenesis, bridging the synaptic cleft and mediating trans-synaptic recognition and signaling [1]. They not only provide a mechanical link but also activate downstream pathways that remodel the cytoskeleton and organize the synaptic apparatus [49,75]. Several key families of synaptic CAMs are involved. Specific molecules, such as SYG-1 and sidekicks, are essential for cellular target recognition, while syndecans regulate synaptic structure and function [76]. In invertebrate models, the Liprin-alpha (syd-2) scaffold is crucial for coordinating cargo trafficking and the structural organization of dendritic spines [77].

Neurexins and neuroligins form heterophilic complexes that act as instructive signaling devices rather than mere structural anchors [12]. These molecules guide synapse formation by specifying postsynaptic receptor composition and release probability through an extensive code of alternative splicing [12]. Regarding elimination, synapse removal is often initiated by the disengagement of these SAM complexes and the cessation of their bidirectional signaling, which secondarily recruits microglia for phagocytic pruning, especially when the ‘synaptic barcode’ fails to validate the connection during activity-dependent competition [1,4,12]. Neurexins, particularly, are expressed in thousands of isoforms due to alternative splicing, contributing to diverse synaptic properties.

Immunoglobulin (Ig)-domain proteins (e.g., SynCAMs): SynCAM1, for instance, promotes the recruitment of GluA1-containing AMPA receptors [27]. Postsynaptic EphBs and ephrin-Bs control multiple aspects of excitatory synaptogenesis, inducing dendritic spine formation and clustering of NMDARs and AMPARs [78]. The homophilic adhesion molecules cadherins are involved in target recognition and synaptic specificity [79].

SPARCL1 (Hevin) is a secreted CAM that boosts excitatory synapse density and enhances AMPAR- and NMDAR-mediated synaptic responses, acting in both synapse formation and shaping [1]. Latrophilins and BAIs are postsynaptic adhesion-GPCRs, critical for initiating synapse formation in specific subsets of synapses, with their engagement activating cytoplasmic signals like cAMP [1].

Synaptic activity plays a crucial role in stabilizing cell adhesion complexes and promoting the expansion of dendritic spine heads, leading to synapse maturation and stabilization [75]. While many structural components are pre-assembled, contact-mediated cell–cell communications and guidance cues (e.g., netrins, FLRTs) are essential for proper wiring and synaptic targeting [80].

### 5.2. Role of Glial Cells in Synapse Development and Pruning

Synaptic pruning is a neurodevelopmental process that refines neural circuitry by systematically eliminating excess or weak synaptic connections, shaping circuit connectivity and function [70]. This process is particularly intense during childhood and adolescence but continues at a reduced pace throughout adulthood [13].

**Figure 6:**
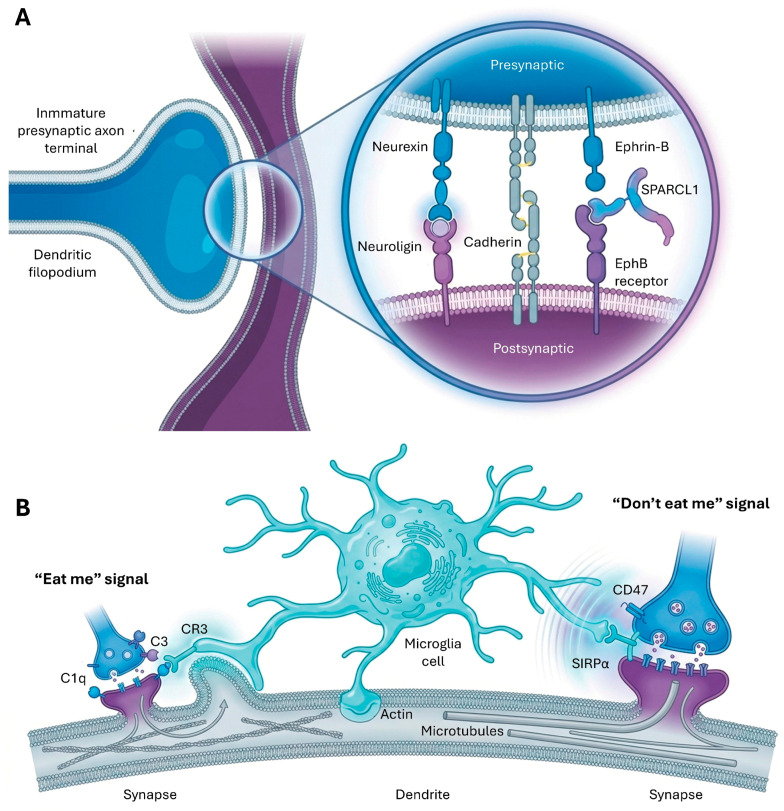
Trans-synaptic adhesion and microglial pruning. (**A**) Synaptogenic cell adhesion molecules (CAMs) bridging the synaptic cleft. Key interactions include Neurexin-Neuroligin, EphrinB-EphB, and Cadherins, which provide both mechanical stability and signaling cues for synapse maturation. (**B**) Synaptic refinement via glial cells. Microglia eliminate weak synapses tagged with “eat me” signals (C1q, C3) through the CR3 receptor, while active synapses are protected by “don’t eat me” signals such as CD47 interacting with SIRPα.

Microglia, the brain’s resident immune cells, play a central role in activity-dependent synapse elimination by engulfing weak or unnecessary synapses [70,80]. It has been discovered that this process strictly requires the activation of caspase-3 within the neuronal postsynaptic compartment. Without caspase activity, microglia fail to recognize and eliminate inactive synapses, which is critical for circuit refinement [73,81]. This process is highly regulated by specific molecular signals. The “eat me” signals include the classical complement cascade, that acts as a primary “tagging” mechanism for microglial pruning [46,81]. Complement proteins, particularly C1q (the initiating protein) and C3, are produced by microglia or astrocytes and localize to apoptotic, immature, or weak developing synapses. These tagged synapses are then recognized and engulfed by microglial complement receptor 3 (CR3). C1q presynaptic tagging correlates with apoptotic markers, guiding pruning decisions [81,82,83]. Other “eat me” signals include phosphatidylserine (PS) exposure on dendrites, recognized by microglial TREM2, and CX3CR1/CX3CL1 signaling, which helps identify synapses for elimination [81,82,83]. Caspase-3 activity is also required for microglia-mediated synapse elimination [73] (Figure 6).

To prevent excessive pruning of healthy, active synapses, neurons express “don’t eat me” signals. CD47, expressed by neurons, binds to its receptor SIRPα on microglia, inhibiting phagocytosis of active synapses [82]. Similarly, the CD200/CD200R pathway (CD200 on neurons, CD200R on microglia) helps protect strong synapses from engulfment [13].

Finally, synapse elimination is an activity-dependent process where weaker inputs are removed while more active ones are strengthened, a process mediated by synaptic competition in postsynaptic cells [46,80,81]. “Punishment signals” propagating from “winner” to “loser” synapses are hypothesized to drive elimination, involving pathways like JAK2-STAT1 in inactive axons and potentially retrograde signals like RhoA or Semaphorins [13].

Astrocytes, another major glial cell type, also contribute significantly to synapse formation and function. They often ensheath synaptic contacts, forming “tripartite synapses” that influence synaptic properties [1]. Astrocytes can secrete synaptogenic proteins, although their precise physiological roles are still being elucidated. Microglia also sense neural activity and modulate neuronal circuits, releasing factors like BDNF to induce new synapse formation or TNFα to regulate synaptic scaling [84].

## 6. Synaptic Diversity and Specialization

The vertebrate nervous system exhibits extensive synaptic diversity, organized into a ‘synaptome’ where functional specialization is driven by the quantitative abundance of shared components rather than the presence of unique proteins [4]. Recent high-yield proteomic profiling using laser-capture microdissection (LCM) across the hippocampal trisynaptic circuit has revealed that this diversity is specifically shaped by the stoichiometry of glutamate receptors and their regulatory partners across microscopic layers (e.g., stratum radiatum vs. stratum lucidum), allowing synapses to assign unique computational properties to specific circuits based on their connectivity [4,5]. This heterogeneity arises from multiple layers of molecular regulation, from gene expression to post-translational modifications.

### 6.1. Molecular Basis of Synaptic Heterogeneity

The proteome of a single synapse is complex, comprising thousands of distinct proteins. This extensive molecular repertoire provides the potential for an almost unlimited number of synapse types, each with unique functional properties [4]. The “synaptome,” which describes the diversity of synapses and their anatomical distribution, is built from the hierarchical expression and assembly of proteins into complexes and supercomplexes [4,10]. MAGUK family scaffold proteins are central to this architecture, linking receptors to signaling cascades; their dysfunction is a convergent factor in neuropsychiatric disorders such as autism and schizophrenia [56,85].

Evolutionary analyses suggest that the increased cognitive power in sophisticated vertebrates is linked to an expansion of synaptic protein diversity, particularly scaffold proteins, which mediate the precise organization of presynaptic release sites and postsynaptic receptors. While some scaffold protein families found in invertebrates are absent in vertebrates, the latter generally possess more isoforms from families such as Dlg, where Dlg2 and Dlg3 have diversified to support complex cognitive functions across vertebrate evolution [7,8].

Synaptic diversity is also evident in subcellular specificity, where specific presynaptic terminals precisely target particular postsynaptic subcellular domains [86]. This specificity can be regulated at various stages of neurodevelopment, including neurogenesis, neuronal migration, axon guidance, and synaptic targeting. The final output of a neuron is determined not only by the type and intensity of synaptic inputs but also by their physical location on the neuron [86].

### 6.2. Role of Alternative Splicing and Post-Translational Modifications

Beyond the sheer number of genes encoding synaptic proteins, alternative splicing and post-translational modifications (PTMs) significantly amplify the molecular diversity of synapses, allowing a single gene to produce multiple protein isoforms (proteoforms) with distinct functions and regulatory properties [87].

Alternative splicing, a process where different combinations of exons are included in the final mRNA transcript, is particularly prevalent in the central nervous system and plays a crucial role in generating synaptic protein complexity [88]. For example, neurexins, key presynaptic CAMs, are expressed in thousands of isoforms due to alternative promoter usage and alternative splicing, with different isoforms exhibiting dramatically varied functions depending on the neuron type [1].

Post-translational modifications (PTMs), such as phosphorylation, palmitoylation, and glycosylation, further diversify the proteome by altering protein function, stability, localization, and interaction partners [5,87]. Recent computational analyses have shown that a significant percentage of PTM sites are excluded from at least one protein isoform due to alternative splicing, and a smaller but notable percentage exhibit altered regulatory sequences surrounding the modification site, suggesting changes in binding interactions [87]. This extensive interplay between splicing and PTMs can rewire protein interaction and kinase-substrate networks, contributing to the fine-tuning of synaptic function and adaptability.

## 7. Integration of Molecular Mechanisms

The molecular physiology of the neuronal synapse is precisely regulated. From the presynaptic active zone’s scaffolding that orchestrates neurotransmitter release to the dynamic postsynaptic density that fine-tunes signal reception, each component contributes to synaptic function. The rapid and efficient recycling of synaptic vesicles ensures sustained neurotransmission, while the precise trafficking and regulation of excitatory and inhibitory receptors dictate synaptic strength and neuronal excitability. Synaptic plasticity, the cellular correlate of learning and memory, emerges from activity-dependent molecular cascades that induce both functional changes in receptor properties and structural remodeling of dendritic spines, often requiring local protein synthesis. Furthermore, the development and refinement of neural circuits are guided by trans-synaptic cell adhesion molecules and the active involvement of glial cells, particularly microglia, in selective synapse elimination. Molecular diversity, amplified by alternative splicing and post-translational modifications, allows for many synaptic specializations, supporting cognitive functions of vertebrates. This integrated molecular machinery underscores the synapse not merely as a point of contact but as an adaptable functional unit.

## 8. Gaps in Current Knowledge and Future Directions

Despite significant progress, several fundamental questions remain unanswered. While various endocytic pathways have been identified, a complete and unified understanding of how synaptic vesicle recycling is controlled as a whole remains elusive [45]. The precise mechanisms that govern the number of vesicles undergoing exo- or endocytosis, ensuring efficient resource allocation and preventing membrane imbalances, are still subjects of active investigation [23]. Future studies should aim to integrate these diverse pathways into a comprehensive, systems-level model. Addressing biological and instrumental variability requires a systems biology perspective, utilizing high-yield procedures such as laser-capture microdissection coupled with deep proteomic profiling. This approach allows researchers to shape a unified model where synaptic diversity is normalized by the levels of core glutamate receptors and their regulatory partners, providing a robust framework across different laboratories [4,5].

The exact nature of the “punishment signals” that propagate from “winner” to “loser” synapses to drive activity-dependent elimination remains largely unknown [46,80,81]. While the involvement of the complement system and microglia is well-established, the precise molecular cues and regulatory mechanisms that tag specific synapses for removal require further elucidation [1].

The totality of specific molecules and chemical signals involved in guiding initial synapse formation and target specificity is not yet completely understood [46,80,81]. Furthermore, how different cell adhesion molecules differentially modulate synaptic specificity and whether all of them act in concert to produce a variety of synaptic connections and strengths needs further investigation [1,89]. The precise role of neurotransmitter signals in synapse establishment, given that abolishing evoked release does not impede ultrastructurally normal synapse formation, presents a significant conundrum [1].

While CaMKII is central to LTP induction, the molecular processes by which the CaMKII/NMDAR complex produces the structural growth of the synapse that underlies late LTP [63]. The stability of the CaMKII-GluN2B complex is proposed as a molecular switch for memory maintenance. This complex may act as a ‘tag’ that captures N-cadherin dimers to increase synapse size and release sites, providing informational stability analogous to DNA base-pairing [53,63]. Future research should focus on identifying the molecular links between initial signaling events and enduring structural changes.

Although alternative splicing and post-translational modifications are known to generate immense synaptic protein diversity, a detailed understanding of how these modifications precisely rewire protein interaction and regulatory networks in specific biological contexts is still emerging [87]. Advanced proteomic and imaging techniques with single-synapse resolution will be crucial for integrating molecular data with cellular functions and circuit organization.

Addressing these gaps will require a combination of experimental approaches, including advanced imaging techniques, high-throughput proteomics, and genetic manipulations in vertebrate models. Computational modeling will also be essential for integrating complex molecular data into predictive frameworks, ultimately informing strategies such as the in vivo conversion of astrocytes into neurons via viral reprogramming, which has shown potential in reversing neuroinflammation and cognitive deficits in Alzheimer’s models [90,91]. Additionally, SV2A PET imaging likely reflects changes in actual synapse density rather than changes in protein levels within remaining synapses [92]. While SV2A levels eventually decrease in correlation with tau accumulation and glymphatic impairment in late-stage Alzheimer’s, certain regions may show a compensatory increase in SV2A binding during early disease stages [93,94,95]. However, parameter estimation and sensitivity analysis in spatial models (reaction-diffusion networks) remain significantly more difficult than in single-compartment models [71]. These models inform strategies like the in vivo conversion of astrocytes into neurons; spatial transcriptomics (Visium) has shown that this conversion can reverse AD-associated signaling patterns, such as the increase in neuregulin signaling between neurons [90,96].

## 9. Conclusions

Neuronal synapses mediate rapid and accurate communication in the brain. This review has described the molecular mechanisms that control presynaptic neurotransmitter release, postsynaptic signal transduction, and the processes underlying synaptic plasticity, formation, maturation, and elimination. At the presynaptic terminal, the active zone organizes vesicle docking and priming through scaffolding proteins such as bassoon, piccolo, RIMs, and munc13. The SNARE complex and synaptotagmin-1 drive calcium-dependent vesicle fusion, followed by vesicle retrieval via clathrin-mediated, kiss-and-run, bulk, and ultrafast endocytic pathways. At the postsynaptic side, the postsynaptic density, structured by scaffold proteins including PSD-95, shank, homer, and gephyrin, regulates the localization and function of excitatory (AMPA, NMDA) and inhibitory (GABA, glycine) receptors, thereby maintaining the balance between excitation and inhibition. Synaptic plasticity, which supports learning and memory, depends on activity-dependent signaling pathways involving kinases (CaMKII, PKA, PKC) and phosphatases (PP1, calcineurin) that modify receptor properties and promote structural changes in dendritic spines, often through local protein synthesis. Neural circuit assembly and maintenance rely on trans-synaptic adhesion molecules, including neurexins, neuroligins, SynCAMs, EphBs, cadherins, SPARCL1, latrophilins, and BAIs, as well as on glial contributions, particularly microglial-mediated synapse elimination through “eat-me” and “don’t-eat-me” signaling. Synaptic diversity, increased by alternative splicing and post-translational modifications, enables functional specialization and adaptive responses required for complex brain functions. Disruption of these molecular mechanisms contributes to the development of neurological and psychiatric disorders such as autism spectrum disorder, schizophrenia, Alzheimer’s disease, and hidden hearing loss (HHL) caused by synaptopathy following GLAST-KO-induced glutamate transporter dysfunction [97].

## Data Availability

No new data were created or analyzed in this study. Data sharing is not applicable to this article.

## Abbreviations

The following abbreviations are used in this manuscript:

AMPA	α-amino-3-hydroxy-5-methyl-4-isoxazolepropionic acid
AMPARs	AMPA receptor
AP2	Adaptor protein-2
AZ	Active zone
Arc	Activity-regulated cytoskeleton-associated protein
BAIs	Brain-specific angiogenesis inhibitors
BDNF	Brain-derived neurotrophic factor
C1q	Complement protein 1q
C3	Complement protein 3
CAMs	Cell adhesion molecules
CAST	Cytomatrix at the active zone-associated structural protein
CAZ	Cytomatrix at the active zone
CB	Collybistin
CD200	Cluster of differentiation 200
CD200R	CD200 receptor
CD47	Cluster of differentiation 47
CME	Clathrin-mediated endocytosis
CR3	Complement receptor 3
CTDs	Cytoplasmic carboxyl termini
CX3CL1	Fractalkine
CX3CR1	Fractalkine receptor
CaMKII	Calcium/calmodulin-dependent protein kinase II
DAPK1	Death-associated protein kinase 1
E-LTP	Early-phase LTP
ERK	Extracellular signal-regulated kinase
EphBs	Ephrin B receptors
F-actin	Filamentous actin
GABA	Gamma-aminobutyric acid
GABAARs	GABA-A receptors
GEF	Guanine nucleotide exchange factor
GKAP	Guanylate kinase-associated protein
GLAST	Glutamate aspartate transporter
GPCRs	G-protein coupled receptors
Geph	Gephyrin
GluA1-4	AMPA receptor subunits
GluN1	NMDA receptor subunit
GluN2	NMDA receptor subunit
GluN3	NMDA receptor subunit
GlyRs	Glycine receptors
HHL	Hidden hearing loss
JAK2	Janus kinase 2
L-LTP	Late-phase LTP
LTD	Long-term depression
LTP	Long-term potentiation
MAGUK	Membrane-associated guanylate kinase
NMDA	N-methyl-D-aspartate
NMDARs	NMDA receptors
NSF	N-ethylmaleimide sensitive factor
NZs	Nascent zones
OCT2	Organic cation transporter 2
PDZ	PSD-95/Dlg/ZO-1
PET	Positron emission tomography
PICK1	Protein interacting with C kinase 1
PKA	Protein kinase A
PKC	Protein kinase C
PP1	Protein phosphatase 1
PS	Phosphatidylserine
PSD	Postsynaptic density
PSD-95	Protein name (specific MAGUK)
PTMs	Post-translational modifications
RIMs	Rab3-interacting molecules
Rab11	Ras-related protein Rab-11
RhoA	Ras homolog family member A
SAP102	Synapse-associated protein 102
SAP97	Synapse-associated protein 97
SER	Smooth endoplasmic reticulum
SERT	Serotonin transporter
SIRP	Signal regulatory protein
SNAPs	Soluble NSF attachment proteins
SNARE	Soluble N-ethylmaleimide sensitive factor attachment protein receptor
SNX27	Sorting Nexin 27
SPARCL1	SPARC-like protein 1 (Hevin)
STAT1	Signal transducer and activator of transcription 1
SV2A	Synaptic vesicle glycoprotein 2A
SVs	Synaptic vesicles
Syb2	Synaptobrevin 2
SynCAMs	Synaptic cell adhesion molecules
SynRIs	Synaptic reuptake inhibitors
Syt1	Synaptotagmin-1
TARPs	Transmembrane AMPA receptor regulatory proteins
TNF	Tumor necrosis factor
TREM2	Triggering receptor expressed on myeloid cells 2
VAChT	Vesicular acetylcholine transporter
VAMP2	Vesicle-associated membrane protein 2
VMAT2	Vesicular monoamine transporter 2
mGluRs	Metabotropic glutamate receptors
mRNAs	Messenger RNAs
t-SNARE	Target SNARE
v-SNARE	Vesicle SNARE
